# The Validity and Reliability of the Persian Version Test of Mobile Phone Dependency (TMD) 

**Published:** 2015-09

**Authors:** Mohammadreza Mohammadi, Seyyed Salman Alavi, Pegah Farokhzad, Fereshteh Jannatifard, Soroush Mohammadi Kalhori, Ghazal Sepahbodi, Mohammad Baba Reisi, Sanaz Sajedi, Mojtaba Farshchi, Rasul Khoda Karami, Vahid Hatami Kasvaee, Neda Sepasi, Samaneh Sadat Alavi

**Affiliations:** 1 Psychiatry and Psychology Research Center, Tehran University of Medical Sciences, Tehran, Iran; 2 Faculty of Psychology, Islamic Azad University of Roudehen Branch, Tehran, Iran; 3 Young Researchers and Elite Club, Roudehen Branch, Islamic Azad University, Roudehen, Iran; 4 Physiology Research Center, Kashan University of Medical Sciences, Kashan, Iran

**Keywords:** *Validity*, *Reliability*, *Mobile Phone Dependency*, *Student*

## Abstract

**Objective:** **‎‏** ‏Despite the fact that ‎the mobile phone has become a ‎pervasive technology of our time, ‎little research has been done on ‎mobile dependency. A valid and ‎reliable assessment instrument ‎corresponding to the Persian ‎culture is essential. This study ‎aimed to describe the ‎construction and validation of the ‎Persian version of TMD (Test of ‎Mobile phone Dependency) to ‎assess the addictive use of ‎mobile phone.‎

**Methods: **This was a cross-‎sectional study, for which data ‎were collected from 350 students ‎who were studying at Tehran ‎universities. Sampling method ‎was quota sampling. The ‎participants anonymously ‎completed the demographic ‎questionnaire, and CPDQ as a ‎valid questionnaire and gold ‎standard. Finally, clinical ‎interview [based on DSM-IV-TR] ‎was performed. To analyze the ‎data, concurrent validity, factor ‎analysis, internal consistency ‎‎(Cronbachα), split half; test-retest ‎and ROC Curve by SPSS18 ‎Software were used.‎

**Results:** As a result of the ‎reliability analysis and factor ‎analysis by principal component ‎and Varimax rotation, three ‎factors (“salient”, “preoccupation” ‎and “Spend a lot of time and ‎money”) for both male and ‎female participants were ‎extracted. Internal consistency ‎‎(Cronbach's alpha) of the TMD ‎was .92 (Cronbach alpha of the ‎factors is .88, .82, and .84, ‎respectively). The test-retest ‎correlation of the TMD was ‎‎.56.The best cut off point for this ‎questionnaire (TMD) is 38.‎

**Conclusion: **The TMD proved to ‎have an acceptable internal ‎consistency with adequate factor ‎models to assess the extent of ‎problems caused by the "misuse" ‎of the mobile phone in the ‎Iranian society. Therefore, it can ‎be concluded that the Persian ‎version of the test was reliable ‎and valid; however, further ‎analysis is needed.‎

Revolution in communication technology, computers, the internet, and mobile phones has led to a dramatic change in the everyday life of the adolescents (1-3).

This is especially true in case of mobile phone which is an important part of the life of the Eastern people (4).The mobile phone is one of the obvious manifestations of the modern world and is an important facility in communication and has become very popular (5).The majority of the Iranian adolescents have a mobile phone. Access to a mobile phone is recognized as a vital necessity among young people in the modern society (6).

Recently, the overuse of Bluetooth has become a common dilemma, and a glance at news and statistics show that such cases are increasing day by day. Also, immoral and absurd messages (SMS) lead to important behavioral abnormality in this context (6).

Researchers and psychologists are apprehensive about the behavioral and psychological aspects that play a role in defining problematic CPU behavior (7).

Problematic CPU could be considered as a form of technological addiction. Because DSM-IV-TR does not offer a category for behavioral addictions (8), confusion has reigned in the diagnosis, treatment and study of these conditions. A popular answer (Griffiths;1996) was given in the work of addiction researchers on non-substance addictions which provided both a bio-psychosocial context and a direction for a comprehensive model of addiction (9, 10, 11).Griffiths (1996) operationally defined technological addiction as a behavioral addiction that involves humane-machine interaction which is non-chemical in nature (9).

History of designing mobile dependency questionnaires has shown that these items have been based on information gathered from the diagnostic criteria for behavioral addiction, CPU addiction etc. Some of these questionnaires are as follows:

**Text-Message Dependency Scale (TMDS):** Igarashi et al. (2008) designed this questionnaire on the basis of the existing studies on text-message use/Young’s criteria for Internet addiction on College students (15-18 years old). It is a15-item questionnaire with Likert spectrum (5 points). The index of reliability, external and internal validity, exploratory and confirmatory factor analyses was assessed. In their study, they extracted emotional reaction, excessive use, and supportive relationship (12).

**PMPUQ Questionnaire**: This questionnaire has been published by Wander linden et al. and measures five main axes about the mobile phone: 1) Questions about mobile phone ownership; 2) Questions about duration of mobile phone ownership; 3) Questions about number of calls in a day; 4) Questions about time of calls; 5) Questions about the number of messages (SMS) in a day. This questionnaire has 30 items and its reliability has been measured by Cronbach alpha (α = 0.85 – 0.90) (13).

**CPDQ Questionnaire**: This 20- item questionnaire has been created by Toda et al., ranging from 1 to 4, with higher scores indicating more phone dependency. One unique characteristic of this questionnaire is shortness, which makes it superior to other questionnaires. A study has reported the reliability of this questionnaire by Cronbach’s alpha (α = 0.80-0.93). Also, validity was calculated by factor analysis; and four factors have been extracted for boys and girls at the high school level (14).

The sharp increase in the use of mobile phones is undeniable. However, its unpleasant and destructive consequences, especially in social and culture fields, should not be neglected. According to social science specialists in Iran which has a large young population many use their mobile phones to send absurd messages and call one another instead of attending to their studies, going to entertaining events or participating in family gatherings (6).

Conducting research on mobile phone is highly regarded by communication and education specialists. Due to the little research done in the fields of mobile phone use, and its psychological effects and considering the few research conducted on the use and abuse of mobile phones, those available valid and reliable questionnaires based on the Iranian culture are fairly restricted to screen the normal mobile phone users from the dependent users. In previous studies done on the reliability and validity of mobile phone questionnaires abroad, defects have been found in the methods (6). Also, we should not accept the reliability and validity of the formal questionnaires. It is necessary to test the instrument that was validated in one culture and adapt it to another population or culture (15).

The present study aimed to examine the psychometric properties of TMD in Tehran University Students. Convergent and concurrent validity of TMD was also examined with its correlations with CPDQ and time of mobile phone use, respectively. The results of this study may allow the psychotherapists and psychologists to have a better understanding of the dimensionality of the construct and applications of mobile phone dependency among adolescents, particularly in students.

## Materials and Method

This was a cross-sectional study, for which stratified sampling was used. Three hundred fifty students participated in the study and completed the questionnaires anonymously. According to all the questionnaires, 72.6% of the participants were female. Inclusion criteria were as follows: Being a student at the time of the study, the participants should have used the mobile phone at least once a day for the past one year; Exclusion criteria were: Severe physical problems or apparent disability, and those who were under treatment due to a specific psychiatric disorder during the past year and were not able to complete the questionnaire. The sample was selected using a stratified sampling method. 

To measure the level of mobile phone dependency, the Persian version of CPDQ, TMD and demographic questionnaire were used. Also, a clinical interview based on DSM-IV-TR criteria for an impulse control disorder (ICD) not otherwise specified (NOS) was conducted.

***Demographic Characteristics:***

The demographic aspects of the questionnaire included gender, age, major and year in college, marital status etc.

***CPDQ (Cellular Phone Dependency Questionnaire)***

It is a 20-item self-report questionnaire and contains questions that reflect typical behaviors of the addicted. It was first used by Toda et al. The participants respond to each item on a 4 -point Likert scale ranging from 1(never) to 4 (always).Possible scores range from a minimum of 20 to a maximum of 80. On the other hand, a higher score indicates a stronger tendency toward cellular phone dependence. The 20-item instrument provides different dimensions of the mobile phone dependency phenomenon. Toda et al. reported that the internal consistency (Cronbach alpha) of the CPDQ was .86, and its test–retest reliability was also reported to be satisfactory (16). Alavi et al. reported that CPDQ proved to be a reliable questionnaire to assess the extent of problems caused by the ‘misuse’ of the mobile phone among the Iranians. In their study, Cronbach alpha of the CPDQ was .88 (Cronbach alpha of the factors were .85, .70, and .76, respectively) (17).

***TMD: Test of Mobile phone Dependency***

These items were constructed according to the criteria contained in DSM-IV-TR for dependence disorder. The initial 101-item questionnaire was reduced to 46 items after a pilot study. The first 18 items were answered on a Likert-type scale ranging from 0 (never) to 4 (frequently). The 28 remaining items asked the respondents to use a Likert-type scale ranging from 0 (completely disagree) to 4 (completely agree) to respond to a set of statements. Six inverse items were included to control the acquiescence effect (18). In this study, we used the brief version (22 items) of the questionnaire.

***Diagnostic Interview***

Clinical interview based on DSM-IV-TR criteria for an impulse control disorder (ICD) not otherwise specified (NOS) was performed for some of the participants by a clinical psychologist who was educated in the diagnosis of ICD, especially in behavior disorder.The interview schedule included items that permit diagnosis with either set of criteria.

To perform this research, the TMD was first translated into Persian by one of the bilingual authors (a native Iranian psychologist translator who was fluent in both English and Persian); then it was edited by a three clinical psychologists who specialized in behavioral addiction. After that, five psychologists and psychiatrists confirmed the content validity of the questionnaire again.

To analyze data, factor analysis (to analyze construct validity), internal consistency (Chronbachα), split half, test-retest and ROC Curve were done by SPSS18 Software.

## Results

The mean age was 21.7 years (SD: 3.5). More than half of the respondents were primarily junior students (51.3%), whereas 48.7% were freshmen or sophomores. Almost one-tenth of the participants (8.5%) were married.

***Analysis of Internal Consistency of the TMD*: **

Demonstrated strong internal consistency estimates. The Cronbach α of the composite test was 0.92, and the item-total correlations of the item ranged from 0.39 (Item 19) to 0.72 (Item 17) (p<0.001). Overall, the results revealed a high level of internal consistency for 22 items, and they were homogenous. Moreover, none of the 22 items had to be deleted to improve α.

***Test –Retest Reliability***

The test–retest reliability of the questionnaire was administered on 50 participants with a one month interval and revealed a significant level of 0.001. Pearson correlation showed a consistency of 0.47 between the two administrations. Also, the split-half reliability of the questionnaire was ascertained with the correlation coefficient between Sets I and II. The correlation was 0.82. Statistical significance was set at 0.05 levels. 

***Validity of the TMD***

***Concurrent Validity***

The correlation between the total scores of TMD and CPDQ revealed a significant positive correlation between the TMD and CPDQ. The correlation between these questionnaires was 0.4, (p<.0001).The moderate effect size of the correlation between TMD and CPDQ provided evidence for the concurrent validity of TMD.

***Convergent Validity***

With respect to the relationship between the TMD scores and time of mobile use, the correlation between the TMD scores and mobile use patterns were also computed. Consistent with the results of Alavi et al. (17), the TMD total was positively related to hours of daily mobile phone use (p<000.1). The proposed diagnostic criteria for mobile addiction consist of excessive time spent on mobile phone and jeopardized work. A significant correlation was found between TMD and daily mobile phone use, providing evidence for the convergent validity of TMD.

***Factor Analysis***

Factor analysis was used to analyze the structure of the questionnaire; principal-components analysis was used to extract the factors and varimax was utilized to perform the rotation. This rotation technique was used because it was assumed that the factors were correlated with one another as the aspects that constitute the component (mobile-phone dependence) are not independent of one another. Some researchers have suggested that if a factor explains 5% of the total variance, the factor is meaningful (19).

The range of factor loadings for the items as well as the Eigen values and variance are shown in Table 1; Eigen values of greater than 1.00 explained variance of 55.48%. The Kaiser–Meyer–Olkin (KMO) index was 0.94 for the adequacy of samples (Bartlett’s test of sphericity was significant, df = 231, P<0.0001) and allowed the rejection of the null hypothesis that the variables used in the analysis were not correlated in the studied population. The Kaiser–Meyer–Olkin (KMO) index suggests that the correlation matrix is suitable for factorization (table 2). All three factors were loaded on expected factors. Factor 1 was the strongest, explaining the greatest percentage of variance (42.59%); fourteen items were loaded on this factor and the factor was named “Salient”.

**Curve 1:**
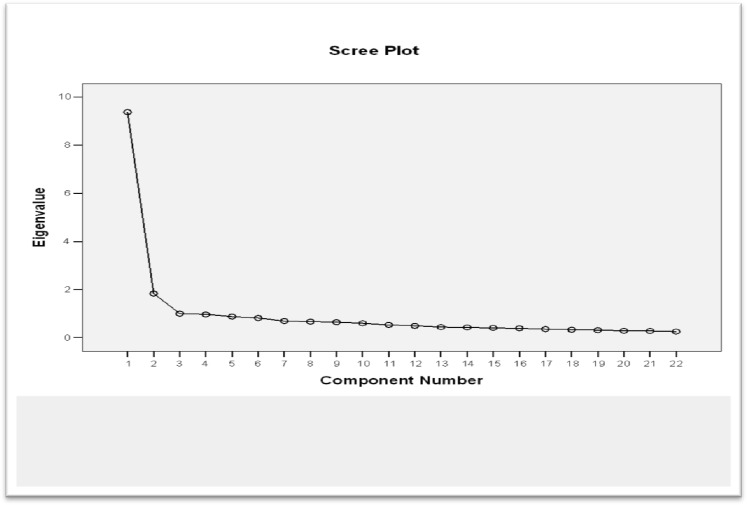
Factors Extracted via Scree Plot

**Table 1:** Total Variance Explained of TMD

**Component**	**Initial Eigenvalues**			**Rotation Sums of Squared Loadings**		
	**Total**	**% of Variance**	**Cumulative %**	**Total**	**% of Variance**	**Cumulative %**
1	9.370	42.590	42.590	5.640	25.637	25.637
2	1.834	8.338	50.928	3.900	17.728	43.366
3	1.001	4.548	55.476	2.664	12.110	55.476

Extraction Method: Principal Component Analysis

**Table 2:** KMO and Bartlett's Test

Kaiser-Meyer-Olkin Measure of Sampling Adequacy		.947
Bartlett's Test of Sphericity	Approx. Chi-Square	3.733
	df	231
	Sig.	.000

**Table 3:** Results of Factor Analysis with Varimax Rotation on 22 Items of TMD

**Items**		**Component**		
		1	2	3
1	I have been called on the carpet or warned about using my mobile phone too much.		.656	
2	I have put a limit on my mobile phone use and I couldn’t stick to it.		.630	
3	I have argued with my parents or family members about the cost of my mobile phone.		.698	
4	I spend more time than I would like to talking on the mobile phone, sending SMSs, or using WhatsApp.		.536	.530
5	I have sent more than five messages in one day.			.744
6	I have gone to bed later or slept less because I was using my mobile phone.			.552
7	I spend more money on my mobile phone (calls, messages…) than I had expected.		.518	.609
8	When I’m bored, I use my mobile phone.	.40		
9	I use my mobile phone (calls, SMSs, WhatsApp...) in situations where, even though not dangerous, it is not appropriate to do so (eating, while other people talk to me, etc.).	.40		
10	I have been criticized because of the cost of my mobile phone.		.767	
11	When I haven’t used my mobile phone for a while, I feel the need to call someone, send an SMS, or use WhatsApp.	.533		
12	Since I got my mobile phone, I have increased the number of calls I make.	.645		
13	If my mobile phone were broken for an extended period of time and took a long time to fix, I would feel very bad.	.762		
14	I need to use my mobile phone more and more often.	.519		
15	If I don’t have my mobile phone, I feel bad.	.755		
16	When I have my mobile phone with me, I can’t stop using it.	.622		
17	Since I got my mobile phone, I have increased the number of SMSs I send	.696		
18	As soon as I get up in the morning, the first thing I do is see who has called me on my mobile phone or if someone has sent me an SMS.	.653		
19	I spend more money now on my mobile phone now than when I first got it.	.40		
20	I don’t think I could stand spending a week without a mobile phone.	.759		
21	When I feel lonely, I use the mobile phone (calls, SMSs, WhatsApp...).	.693		
22	I would grab my mobile phone and send a message or make a call right now.	.552		

Extraction Method: Principal Component Analysis.

Rotation Method: Varimax with Kaiser Normalization.

Six items that reflected factor 2 included the following items: 1, 2, 3, 4, 7 and 10; this factor was named “Preoccupation”. Four items of the TMD were loaded on factor 3, which was named “Spend a lot of time and money” (Table3).

In order to measure internal consistency within the items in each factor, Cronbach alphas were calculated and all were moderately to highly reliable (0.73–0.92). In addition, the factor analysis provided support for the construct validity of the questionnaire.

***Determination of the Cut of Point***

A total of 80 participants (22.8%) were identified as having mobile phone dependency by the systemic diagnostic interview based on the DSM-IV-TR, and were diagnosed as having behavioral addiction, especially mobile phone addiction. In this study, the gold standard chosen was the psychological interview. 

All participants were further divided into screening positive (n = 80) and screening-negative groups.

Clinician-administered schedules based on DSM-IV-TR are often considered as the gold standard in epidemiological researches. The ROC (Receiver Operating Characteristic curve) analysis for the TMD gave an area under the curve of 95%, indicating that the TMD had good diagnostic efficiency (CI =. /92-. /97). The cut-off point of 38 was best for discriminating cases of mobile dependency from non-cases, with a high diagnostic accuracy (95%), and specificity (89%). A TMD cut-off point of 38 resulted in a high sensitivity (93%) and an acceptable specificity (89%), showing that this was an optimal cut-off point to screen the possible cases of mobile dependency.

## Discussion

Although a growing number of studies have assessed the severity of mobile use problems in adolescents, no Iranian studies have established the factorial structure of TMD in this population for psychologists’ references. The present study has explored the dimensionality of TMD, and evaluated its psychometric properties in students of Tehran universities. 

The sampling method was based on inclusion and exclusion criteria, and the specialists' diagnosis clarified the cut-off point by comparing the mobile phone dependents and non-dependents via ROC analysis. 

Three types of reliability were used to assess tests, scales and questionnaires. Reliability results by Cronbach’s alpha was 0.92 (factor 1 α = 0.88, factor 2 α = 0.82, factor 3 α = 0.84, respectively), it was 0.82 by split-half, and0.47 by test-retest technique. Follow up study was done on 50 students after one month; they significantly used mobile phone; therefore, the results revealed good reliability by previous studies. Coliz in a study reported Cronbach’s alpha of the Spanish version of this questionnaire to be 0.94 (18). Another study reported acceptable psychometric properties for mobile phone problem use scale (20). Also, subscales had high internal consistency with integrative items. Many researches indicated that Cronbach’s alpha coefficients (the internal reliability) of the items were further supported by the strength of the total item correlations which exceeded the 0.7 recommended threshold (21).Therefore, internal consistency by Cronbach’s alpha and other methods of reliability assessment presented acceptable results for the Persian and original version of the TMD questionnaire. 

Moreover, the results revealed the same consistency and problems related to the Iranian culture in comparison to the initial form of TMD. 

TMD has a relationship with other mobile phone dependency scales. One of the assessments of mobile phone self-reported scale is CPDQ. There was an acceptable correlation between TMD and CPDQ scores (r = 0.40, p< 0.01). Also, the total scores of the questionnaire and mobile phone usage time showed a significant and positive relationship(r = 0.38, p<0.0001). Coliz in his study conducted on 347 adolescents (51.6% girls and 48.2% boys), evaluated the construct validity of TMD by correlating it with the Mobile Phone Dependence Questionnaire (MPDQ) (18). Alavi et al. also reported that concurrent validity of the mobile phone addiction questionnaire was assessed by the relationship with time per day spent on using a mobile phone (17). Therefore, the significant and positive relationship between the total score of the questionnaire and the time spent on mobile phone proves the concurrent validity.

The results of the factor analyses revealed that the TMD can be characterized by exploring three factors of mobile addiction: “Salient”, “Preoccupation” and “Spend a period of time and money”. Also, Coliz (2012) found three factors which were extracted for 12-18 year old adolescents, which were similar to this study (18). In addition, behavioral addictions which were assessed by experts using six factors were highly similar to these factors. For example, many experts like Brown discussed and explored the concept of addiction with six criteria: Salience: Domination of a person’s life by the activity; Euphoria: A ‘buzz’ or a ‘high’ is derived from the activity;Tolerance: The activity has to be undertaken to a progressively greater extent to achieve the same ‘buzz’;Withdrawal Symptoms: Cessation of the activity leads to the occurrence of unpleasant emotions or physical effects;Conflict: The activity leads to conflict with others or self-conflict; and Relapse and Reinstatement: Resumption of the activity with the same vigor subsequent to attempt to abstain negative life outcomes and negligence of job, educational or career opportunities (22).Therefore, the Persian version of TMD forms the behavioral addiction based on three main dimensions of mobile phone problem use, showing inner relationship dependency in the general and clinical population. 

In sum, explanatory factor analysis validates the TMD as a questionnaire measuring three different facets associated with problematic mobile phone use. This questionnaire which has reasonable psychometric properties assesses the followings: 1) Preoccupation with mobile phone (14 items); (2) Spending a lot of time and money (4 items); (3) Salience (4 items). Three psychometric properties of discriminative analysis such as cutoff point, sensitivity (0.93) and specificity (0.89) are the best subscales for differential diagnostic procedures between cell-phone dependents and non-dependents. 

Also, the cutoff point of this tool has suitable sensitivity (0.93) for discriminating dependent and non-dependent mobile phone users. In sum, the questionnaire had a high and great specificity. These results revealed that the screening and diagnostic cut-off points can identify higher frequency users efficiently. However, previous studies showed that correlation coefficient remained the same with the primary and original questionnaire. Also, other researches in different countries had the same results (18). This finding demonstrated that this scale has unique characters for the Persian and English languages and contains fluent and comprehensive phrases, and it is an ideal assessment tool to be used in the cultural context of Iran. 

## Limitations and suggestions

This study had several limitations. At first, although data on concurrent validity were presented in relation to the main parameters of the use of mobile phones, additional analyses are necessary to examine the associations between the scale and mental health conditions (for example: depression, anxiety) and other indicators of psychosocial dysfunction. However, the main problem was related to the impressive growth of technological functions and applications of the mobile phone (23, 24). It is of prime importance to conduct studies on conditions that foster this dependence to develop prevention and treatment programs and make available assessment and diagnostic instruments that enable effective intervention.

## Conclusion

مطالعه کنونی نشان می دهد که پزشکان و پرستاران تمایل بیشتری دارند تا تشخیص سرطان را با بیمار در میان بگذارند.This study has provided an empirical foundation of the psychometric properties of the assessment of Mobile Phone Dependency, which could benefit future researches on the life-course risks of the mobile phone.

Consistent with a previous study on Spanish adults, a higher order latent factor was satisfactorily accounted by three underlying factors. Therefore, TMD is a valid and reliable questionnaire for identifying pathological mobile phone use among Iranian adolescents. Validation of the Persian TMD in adolescents is important for the early detection in clinical practice and conducting multiethnic epidemiological studies in research settings. ***محدودیتها و پیشنهادات***
